# Pexidartinib: Current advances in the symptomatic treatment of tenosynovial giant cell tumors

**DOI:** 10.1007/s10555-026-10328-z

**Published:** 2026-04-02

**Authors:** Yulin Yuan, Zhuoer Zhang, Yanfeng Jiang, Ting Ye, Jing Chen

**Affiliations:** 1 Cancer Center, Union Hospital, Tongji Medical College, Huazhong University of Science and Technology, Wuhan, Hubei China; 2 Hubei Key Laboratory of Precision Radiation Oncology, Wuhan, Hubei China; 3 Department of Bone and Soft Tissue Tumors, Affiliated Hospital of Jiangxi University of Chinese Medicine, Nanchang, Jiangxi China; 4 Institute of Radiation Oncology, Union Hospital, Tongji Medical College, Huazhong University of Science and Technology, Wuhan, Hubei China

**Keywords:** Pexidartinib; Tenosynovial Giant Cell Tumor; Colony﻿-Stimulating Factor 1 Receptor

## Abstract

Tenosynovial giant cell tumors (TGCTs), a rare benign mesenchymal neoplasm of synovial tissue, often incurs chronic pain, joint destruction and repeated surgery, markedly impairing quality of life. Historically, surgery was the only effective option. The colony-stimulating factor 1 receptor (CSF1R) inhibitor pexidartinib broke this therapeutic deadlock, pioneering systemic therapy and shaping subsequent drug development. Further exploration of pexidartinib and associated clinical studies in the field of TGCT have continued following its approval, with updated data and outcomes continuing to play a crucial role in guiding the clinical application of pexidartinib for treatment of TGCT. This review provides a comprehensive summary of the preclinical and clinical development of pexidartinib for treatment of TGCT, and highlights the recent updates in clinical studies and findings since its approval. These include long-term efficacy, optimization of therapeutic strategies, management of risks associated with long-term use, and real-world patient-reported outcomes, all of importance and value for patients and physicians in clinical practice. Here we aim to provide guidance for the improved clinical application of this drug class to enhance patient benefits in TGCT treatment.

## Introduction

Tenosynovial giant cell tumors (TGCTs) are rare, typically nonmalignant, locally aggressive proliferations of synovial membranes surrounding joints and tendons [1]. Clinically, TGCT primarily presents with pain and swelling, accompanied by stiffness, reduced range of motion, and joint instability, with slow disease progression [2, 3], and is classified into localized (nodular) and diffuse subtypes: localized TGCT manifests as a single defined tumor often in smaller joints including the fingers and toes, while diffuse TGCT is extensive and infiltrative, typically affecting larger joints such as the knees and extending into extra-articular structures [1, 4, 5]. The clinical spectrum of TGCT is broad, with variability in symptom experience between patients, and can be associated with substantial morbidity [1, 2]. Although not life-threatening, patients may experience long-term symptoms and functional impairment due to joint destruction and recurrent surgical interventions, significantly affecting their daily activities and quality of life [1, 2, 6].

TGCT is typically diagnosed in patients approximately 35–50 years old, and more commonly affects female than male patients (male:female ratio: up to 1:1.5) [1, 7–9]. The relatively young age at diagnosis places a substantial burden on both patients and the wider healthcare system, with a substantial deterioration of health-related quality of life and associated treatment costs over time identified in studies conducted in the US and Europe [6, 10–12]. The global incidence rate of TGCT has been estimated at 43 cases per million people per year (29, 10, and 4 per million people per year for digital, nodular [localized], and diffuse TGCT, respectively) based on studies conducted in The Netherlands (2009–2014) and Denmark (1997–2012) [4, 7, 13].

Surgery (synovectomy) remains the primary treatment strategy for patients with symptomatic TGCT [1, 14]. However, recurrence of TGCT after surgery is common, particularly in those with diffuse disease: 23–72% of patients with diffuse TGCT experience recurrence following surgery [1, 7, 15, 16], which can also be accompanied by long-term impairments in function and quality of life, in addition to risks of postoperative complications, deformity and disability [6, 17, 18]. While the mechanisms underlying TGCT recurrence remain incompletely understood [19], proposed risk factors include inadequate marginal excision, bone invasion, proximity to interphalangeal joints, and Al Qattan type 2 (at least two spatially isolated) tumors [20, 21]. There is some evidence to support the use of radiotherapy for treatment of TGCT, used with the aim of reducing the risk of recurrence following surgical resection as well as in neoadjuvant and relapsed settings [22–27]. However, the use of radiotherapy in this setting remains controversial, since research to date consists predominantly of small retrospective analyses in which results appear mixed, with a recent expert consensus noting insufficient data to support the use of radiotherapy as a standard treatment [1]. Patients with difficult-to-manage TGCT who are not amenable to surgery, or those facing recurrent disease following surgery, are therefore left with limited treatment options [1]. For a long time, TGCT treatment lacked effective therapeutic options beyond surgery, until the successful development and commercialization of colony-stimulating factor 1 receptor (CSF1R) inhibitors.

In 2006, West et al. identified colony-stimulating factor 1 (CSF1) as a key driver of TGCT by describing the paracrine “landscape effect” [28]. Since CSF1 is involved in the proliferation and function of macrophages, CSF1 overexpression by a small percentage of neoplastic synovial-lining cells leads to the recruitment of non-neoplastic chronic inflammatory cells, including CSF1R-expressing macrophages, creating a tumor “landscape” that results in the hyperplasia of synovial cells around joints and tendon sheaths [28, 29, 32]. CSF1 overexpression is a key hallmark of TGCT, driven by a range of somatic mutations and alternative mechanisms including (1;2)(p13;q37) translocation on chromosome 1p13 corresponding with the *CSF1* gene leading to fusion of *CSF1* and *COL6A3* in approximately 30% of patients [1, 28–31, 33]. CSF1 overexpression is often observed irrespective of *CSF1* fusion [1], with alternative factors associated with CSF1 upregulation including other fusion variants that may not involve *CSF1*, *CSF1* exon 9 deletion and 3’ truncation, trisomy 5 and/or 7, p53 mutations, and *CBL* missense mutations [5, 29, 33–35].

Given these advances in the understanding of TGCT as both a neoplastic process involving CSF1 overexpression as well as inflammatory pathways [28, 36], the CSF1/CSF1R axis has been investigated as a molecular target for systemic therapy [37]. Pexidartinib was the first CSF1R inhibitor approved for the treatment of TGCT in 2019, with studies demonstrating tumor shrinkage, symptomatic and functional improvement, and long-term disease control [38], marking a significant breakthrough in the systemic treatment of TGCT [39–41]. This approval in the US was through a Risk Evaluation and Mitigation Strategy (REMS) program, a Food and Drug Administration (FDA)-mandated drug safety program. It was not until 2025 that the second CSF1R inhibitor for TGCT, vimseltinib, received US approval [42, 43]. Pexidartinib therefore serves as a pioneer in the systemic treatment of TGCT, with its development process and clinical study design laying the groundwork and providing direction for subsequent advancements. Pexidartinib has since been approved in South Korea (2021), and Taiwan (China, 2022) for treatment of adults with symptomatic TGCT with severe morbidity or functional limitations who are not amenable to improvement with surgery (or other treatment in Taiwan) [44, 45]. Following a phase 3 bridging study in the Chinese mainland and Taiwan (China) [46], pexidartinib is also undergoing priority review for TGCT by the Center for Drug Evaluation (CDE) of the Chinese National Medical Products Administration (NMPA).

While reviews to date have predominantly focused on the period after the US approval of pexidartinib from 2020–2022 [14, 15, 47–50], comprehensive reviews summarizing more recent data are lacking. Here, we consolidate the current and emerging clinical evidence for systemic treatment with pexidartinib in patients with TGCT, with a focus on studies that provide clinically meaningful insights for the application of pexidartinib after its market launch to enhance patient benefits. These include long-term efficacy, optimization of therapeutic strategies, management of risks associated with long-term use, and real-world patient-reported outcomes, all of importance and value for both patients and physicians in clinical practice.

## Pexidartinib preclinical development

Preclinical and clinical studies demonstrated that pexidartinib interacts with the juxtamembrane domain of CSF1R to stabilize and maintain its autoinhibitory state, inhibiting the binding of CSF1 and ATP and so preventing ligand-induced auto-phosphorylation [47, 51]. Pexidartinib potently inhibits CSF1R (biochemical half maximal inhibitory concentration [IC_50_]: 0.013 μM) and c-KIT (KIT; IC_50_: 0.027 μM) receptor tyrosine kinases with significantly higher potency than conventional type 2 inhibitors (e.g., imatinib, IC_50_: 0.67 μM [CSF1R] and 0.21 μM [KIT]), and has demonstrated limited cross-reactivity with other kinases (> 0.1 μM for CDK19, FLT3, KDR, LCK, FLT1, and TRK3, and > 1 μM for all other kinases tested) [51].

In TGCT cell lines, pexidartinib inhibits cell growth and induces tumor cell apoptosis, with responses that correlate with the level of CSF1R expression [52]. In addition, a modulatory effect of pexidartinib on macrophages and T cells correlating with suppression of tumor growth was identified in an osteosarcoma orthotopic xenograft model, involving suppression of pERK stimulation by CSF1, and depletion of tumor-associated macrophages and FOXP3+ T cells [53]. In an experimental model of TGCT (mouse collagen-induced arthritis), pexidartinib (10 mg/kg) was found to significantly delay the progression and reduce the severity of bone erosion, cartilage destruction, synovitis and pannus formation with an inhibitory effect on osteoclast marker cathepsin L and macrophage marker F4/80 [51]. Pexidartinib has also exhibited desirable pharmacokinetics in mice, rats, dogs, and monkeys, with low systemic clearance, high volume of distribution, and oral bioavailability > 40%, with dose-proportional increases in systemic exposure [51]. Together, these results were suggestive of a pharmacological effect of pexidartinib in human joint-related diseases.

## Clinical efficacy and safety of pexidartinib

### Phase 1 studies

The safety, pharmacokinetics, and tumor response to pexidartinib were first assessed in a phase 1 dose-escalation trial [51]. Pexidartinib had an acceptable safety profile and was generally well tolerated among 41 patients with solid tumors driven by CSF1R, KIT, or FMS‐like tyrosine kinase 3 (FLT3) that were refractory to standard therapy, and a maximum tolerated dose (and chosen phase 2 dose) of 1,000 mg/day was established [51]. In the extension phase of the study among 23 patients (data cutoff: April 2014), 12 patients with TGCT had a partial response, and 7 had stable disease, with an objective response rate (ORR) of 52%, disease control rate (DCR) of 83%, and median duration of response (DOR) of > 8 months (Table 1) [51]. The most common treatment-emergent adverse events (TEAEs) were changes in hair color (73.9%), fatigue (78.3%), and nausea (65.2%; Table 2). The majority of treatment-related events were mild to moderate in severity, and 2 patients discontinued study treatment due to AEs. Grade ≥ 3 treatment-related adverse events (TRAEs) reported in 8 (34.8%) patients included fatigue, diarrhea, anemia, hyponatremia, elevated liver enzymes (aspartate aminotransferase [AST] or alanine aminotransferase [ALT]), and neutropenia. These findings were further supported by the subsequent phase 1 extension study (interim data cutoff: January 2018), which further evaluated the efficacy, safety, and pharmacokinetics of pexidartinib among 91 patients with six different solid tumors including 39 patients with TGCT [54]. The median progression-free survival (PFS) among patients with TGCT was not reached (95% confidence interval [CI]: 667 days–not applicable [NA]), the ORR was 62% as assessed by Response Evaluation Criteria in Solid Tumors (RECIST) v1.1, and 56% by tumor volume score (TVS, a TGCT-specific measure of tumor reduction that calculates tumor volume as a proportion of the distended synovium, providing a more accurate quantification of response in TGCT lesions that are often irregular in shape [51], compared with linear methods such as RECIST [55]) among patients with TGCT [54]. Pain improvements from baseline by self-reported numeric rating scale (NRS) were also observed in patient-reported outcomes [54].

**Table 1:** Overview of pexidartinib efficacy results

Trial or study	Phase	Patient N	Intervention	Study population	Efficacy outcomes
PLX108-01 TGCT cohort [51] (NCT01004861; dose-extension)	1	23	Pexidartinib (PLX3397; 1000 mg/day)	Patients with TGCT	• ORR: 52% • DCR: 83% • Median DOR: > 8 months • Median PFS: not reached
PLX108-01 TGCT cohort [54] (NCT01004861; part 2, extension)	1	39	Pexidartinib (1000 mg/day)	Patients with TGCT	• ORR (FAS): 62% (RECIST); 56% (TVS) • DOR (25th percentile): 943 days • DCR (FAS): 82% (RECIST); 79% (TVS) • Median PFS: not reached
PL3397‐A‐A103 [101] (NCT02734433)	1	11 (1 patient with TGCT)	Pexidartinib 600 mg/day (cohort 1) or 1000 mg/day for two weeks, then 800 mg/day (cohort 2)	Asian patients with symptomatic, advanced solid tumors (including TGCT)	• ORR: 13% (RECIST) – the patient with TGCT had a PR and saw a large decrease in lesion diameter at 7 months that was sustained to study cutoff • DCR: 63%
ENLIVEN, part 1 [38] (NCT02371369)	3	120 (pexidartinib n = 61 vs placebo n = 59)	Pexidartinib (1000 mg/day for two weeks, then 800 mg/day) versus placebo	Patients with symptomatic, advanced TGCT not amenable to surgery	• ORR at Week 25: 39% vs 0% (RECIST; 95% CI: 27–52%; p < 0.0001); 56% vs 0% (TVS; 95% CI: 42–68%; p < 0.0001) • ORR (January 31, 2018 data cutoff): 53% (RECIST); 64% (TVS) • CfB in ROM (Week 25): + 15% vs + 6% (p = 0.0043) • CfB in PROMIS (Week 25): + 4.1 vs − 0.9 (p = 0.0019) • CfB in worst stiffness (Week 25): − 2.5 vs − 0.3 (p < 0.0001) • CfB in pain score (Week 25): − 2.5 vs − 0.6 (p < 0.0001)
ENLIVEN, part 2 [38] (NCT02371369)	3	30	Pexidartinib (800 mg/day); patients received placebo to Week 25 in part one and crossover to pexidartinib in part two open-label	Patients with symptomatic, advanced TGCT not amenable to surgery initially randomized to placebo in part 1 who entered part 2 and received open label pexidartinib treatment	• ORR at Week 25: 30% (RECIST); 57% (TVS) • ORR (January 31, 2018 data cutoff): 53% (RECIST); 67% (TVS) • CfB in ROM (Week 25): + 13% • CfB in PROMIS score (Week 25): + 4.9 • CfB in stiffness score (Week 25): − 3.0 • CfB in pain (Week 25): − 2.6
ENLIVEN, final results [60]	3	91	Pexidartinib (1000 mg/day for two weeks, then 800 mg/day) versus placebo	Patients with symptomatic, advanced TGCT not amenable to surgery enrolled in the phase 3 ENLIVEN study	• ORR (Week 240 data cutoff April 2021): 60% (RECIST), 68% (TVS) • Median DOR: not reached
Xu et al. 2025 [58] (NCT04488822)	3	40	Pexidartinib (800 mg/day)	Chinese patients with TGCT and severe morbidity/functional limitations not amenable to surgery (mainland China and Taiwan)	• ORR at Week 25: 22.5% (RECIST); 47.5% (TVS) • ORR (Week 37 data cutoff December 21, 2021): 30.0% (RECIST); 47.5% (TVS) • CfB in ROM (Week 25): + 23.1 degrees
Desai et al. 2024 [75] (NCT04526704)	4	32	Pexidartinib (≥ 1 dose)	Patients with TGCT experiencing clinical benefit from pexidartinib in one of four prior phase 1 or 3 studies	• Treatment continuation cohort: no patients experienced PD over 24-month study period • Treatment-free/retreatment cohort: 54.5% had PD (RECIST), median PFS: 22.8 months, 73% remained treatment-free at 12 and 24 months • PROMIS and EQ-5D-5L VAS scores remained stable over time • 45% of patients who stopped taking pexidartinib did not exhibit PD by RECIST
Gelderblom et al. 2021 [62]	Pooled analysis	130	Pexidartinib (1000 mg/day, or 1000 mg/day then 800 mg/day)	Patients with symptomatic, advanced TGCT not amenable to surgery	• ORR: 60% (RECIST), 65% (TVS) • Median DOR: not reached (RECIST), 46.8 months (TVS)
Healey et al. 2023 [63]	3 (exploratory analysis of pain outcomes)	120	Pexidartinib 1000 mg/day for two weeks, then 800 mg/day) versus placebo	Patients with symptomatic, advanced TGCT not amenable to surgery enrolled in the phase 3 ENLIVEN study	• ≥ 30% improvement in worst pain: 31% vs 15% (p = 0.03) • ≥ 50% improvement in worst pain: 26% vs 10% (p = 0.02) • MCID in worst pain:^a^ 31% vs 14% (p = 0.02) • CfB in worst pain score (Week 25, part one): − 2.5 vs − 0.3 (p < 0.001) • CfB in worst pain score (Week 25, part two): − 2.7 • CfB in worst pain score (Week 50, part two): − 3.3
Healey et al. 2024 [80]	3 (exploratory analysis according to surgical history)	120	Pexidartinib 1000 mg/day for two weeks, then 800 mg/day) versus placebo	Patients with symptomatic, advanced TGCT not amenable to surgery enrolled in the phase 3 ENLIVEN study (prior surgery vs no prior surgery cohorts)	• ORR at Week 25: 34.4% in those with prior surgery versus 41.4% in those without (RECIST; 53.1% vs 55.2% by TVS) • ORR at study completion (open-label extension 25 weeks to 5.5 years): 56.3% in those with prior surgery versus 65.5% in those without (RECIST; 68.8% vs 65.5% by TVS) • Three patients were able to undergo post-treatment surgery due to reduced morbidity and improved probability of complete resection
Lin et al. 2024 [65]	Real-world observational study	83	Pexidartinib (≥ 1 dose)	Patients with TGCT in the US REMS program not previously enrolled in a clinical trial for pexidartinib	• Joint symptoms (pain, stiffness, joint sound during movement, limited range of motion, warmth of the skin over the joint and swelling): improved in 78.3% of patients (27.7% “Very much improved”, 30.1% “Much improved”, 20.5% “Minimally improved”) • Physical function: improved in 77.1% of patients (30.1% “Very much improved”, 28.9% “Much improved”, 19.3% “Minimally improved”) • Stiffness: 73.2% of patients reported ≥ 1-point improvement, 65.9% reported ≥ 2-point improvement • Worst stiffness NRS: 3.0 with pexidartinib versus 6.2 before pexidartinib (p < 0.001) • Worst pain NRS: 2.7 with pexidartinib versus 5.7 before pexidartinib (p < 0.001)
Dai et al. 2023 [67]	Real-world observational study	31	Pexidartinib (≥ 1 dose)	Patients with TGCT enrolled in US REMS program	• Physical function: improved in 59.0% of patients, mean change in PROMIS score of − 0.43 from first dose to follow-up (not significant) • CfB in worst stiffness NRS score: − 3.2 (0.8 from first dose to follow-up, not significant) • CfB in worst pain NRS score: − 3.0 (− 0.1 from first dose to follow-up, not significant)
Dai et al. 2024 [66]	Real-world observational study	11	Pexidartinib (≥ 1 dose)	Patients with TGCT enrolled in US REMS program who newly initiated pexidartinib treatment	• Moderately high satisfaction: 72.7% of patients • Overall symptom improvement: 87.5% of patients • Average rate of change (ARoC) in worst pain score: − 2.44 • ARoC in worst stiffness score: − 1.82 • ARoC in improvement in PROMIS score: 6.33
Bernthal et al. 2022 [76]	Case series	3	Pexidartinib + surgery	Patients with previously unresectable or inoperable TGCT	• Case 1: pexidartinib was associated with tumor reduction prior to surgery, with no recurrence at 12 months post-surgery • Case 2: pexidartinib was associated with tumor reduction following post-surgery recurrence within 4–5 months of treatment, and sustained symptom control • Case 3: pexidartinib pre- and post-surgery was associated with sustained disease control
Geiger et al. 2024 [77]	Case study	1	Pexidartinib 800 mg/day post-surgery	Patient with diffuse TGCT recurrence after surgery	• Improvements in tumor size and pain within one month that facilitated further surgery • Pexidartinib + further surgery was associated with improvements in motility and return to work
Giustini et al. 2018 [79]	Case study	1	Pexidartinib 1000 mg/day	Patient with diffuse TGCT not amenable to surgery	• Tumor volume decreased by 48% after 4 months of treatment • Continued disease stability observed after 55 months of treatment
Palmerini et al. 2024 [78]	Case study	1	Pexidartinib 800 mg/day, then dose reductions to 400 mg/day and 200 mg/day	Patient with diffuse TGCT not amenable to surgery	• CR achieved, pain reduced, and mobility restored after two years of treatment

^a^The threshold for MCID was defined as a decrease in worst pain NRS score ≥ 50% with a decrease of at least two points [63, 64]. ARoC, average rate of change; CfB, change from baseline; CI, confidence interval; CR, complete response; DCR, disease control rate; DOR, duration of response; EQ-5D-5L, EuroQol 5-dimension, 5-level scale; FAS, full analysis set; MCID, minimal clinically important difference; NRS, numeric rating scale; ORR, objective response rate; PFS, progression-free survival; PR, partial response; PROMIS, Patient-Reported Outcomes Measurement Information System − Physical Function scale; RECIST, Response Evaluation Criteria in Solid Tumors; REMS, risk evaluation and mitigation strategy; ROM, range of motion; SD, stable disease; TGCT, tenosynovial giant cell tumor; TVS, tumor volume score; VAS, visual analog scale

**Table 2:** Overview of pexidartinib safety results

TEAE, n (%)	PLX108-01 TGCT cohort [51] (1000 mg/day) n = 23	PLX108-01 extension TGCT cohort [54] (1000 mg/day) n = 39	ENLIVEN, part 1 [38] (1000 mg/day for two weeks, then 800 mg/day) n = 61	ENLIVEN, part 2^a^ [38] (800 mg/day) n = 30	ENLIVEN, final results [60] n = 91	Xu et al. 2025 [58] (800 mg/day) n = 40	Desai et al. 2024 [75] n = 24
All TEAEs	23 (100.0)	39 (100.0)	60 (98.4)	NR	91 (100)	40 (100.0)	22 (91.7)
Discontinuation	2 (8.7)	NR	8 (13.1)	NR	18 (19.8)	9 (22.5)	2 (8.3)
Deaths related to study treatment	0 (0.0)	NR	0 (0.0)^b^	NR	0 (0.0)	0 (0.0)	0 (0.0)
Most common TEAEs (≥ 25% of patients,^c^ any grade)							
Skin disorders							
Change in hair color	17 (73.9)	28 (71.8)	41 (67.2)	25 (83.3)	69 (75.8)	31 (77.5)	NR
Pruritus	NR	14 (35.9)	10 (16.4)	8 (26.7)	19 (20.9)	19 (47.5)	NR
Rash	NR	12 (30.8)	8 (13.1)	6 (20.0)	25 (27.5)	17 (42.5)	NR
Gastrointestinal disorders							
Nausea	15 (65.2)	26 (66.7)	23 (37.7)	6 (20.0)	35 (38.5)	6 (15.0)	3 (12.5)
Diarrhea	7 (30.4)	14 (35.9)	12 (20.0)	8 (26.7)	29 (31.9)	5 (12.5)	NR
Vomiting	7 (30.4)	12 (30.8)	12 (20.0)	1 (3.3)	19 (20.9)	NR	4 (16.7)
Constipation	NR	11 (28.2)	7 (11.5)	3 (10.0)	NR	4 (10.0)	NR
General disorders							
Fatigue	18 (78.3)	36 (92.3)	33 (54.1)	5 (16.7)	42 (47.3)	12 (30.0)	3 (12.5)
Face edema	NR	NR	8 (13.1)	6 (20.0)	NR	17 (42.5)	NR
Investigations							
AST increased	NR	NR	24 (39.3)	5 (16.7)	34 (37.4)	23 (57.5)	6 (25.0)
ALT increased	NR	NR	17 (27.9)	7 (23.3)	26 (28.6)	24 (60.0)	NR
LDH increased	NR	NR	7 (11.5)	3 (10.0)	NR	19 (47.5)	NR
GGT increased	NR	NR	NR	NR	NR	15 (37.5)	NR
White blood cell count decreased	NR	NR	NR	NR	NR	14 (35.0)	NR
ALP increased	NR	NR	9 (14.8)	1 (3.3)	NR	12 (30.0)	NR
CPK increased	NR	NR	NR	NR	NR	8 (20.0)	10 (41.7)
Neutrophil count decreased	NR	NR	NR	NR	NR	11 (27.5)	NR
Nervous system disorders							
Dysgeusia	6 (26.1)	14 (35.9)	15 (24.6)	7 (23.3)	25 (27.5)	4 (10.0)	NR
Dizziness	6 (26.1)	11 (28.2)	6 (9.8)	4 (13.3)	NR	4 (10.0)	NR
Headache	NR	13 (33.3)	11 (18.0)	5 (16.7)	21 (23.1)	NR	NR
Musculoskeletal disorders							
Arthralgia	9 (39.1)	24 (61.5)	14 (23.0)	6 (20.0)	34 (37.4)	4 (10.0)	4 (16.7)
Pain in extremity	6 (26.1)	10 (25.6)	4 (6.6)	3 (10.0)	NR	NR	NR
Eye disorders							
Periorbital edema	6 (26.1)	15 (38.5)	11 (18.0)	3 (10.0)	22 (24.2)	9 (22.5)	NR
Metabolic/nutritional disorders							
Decreased appetite	6 (26.1)	9 (23.1)	10 (16.4)	3 (10.0)	NR	4 (10.0)	NR
Vascular disorders							
Hypertension	NR	8 (20.5)	9 (14.8)	6 (20.0)	26 (28.6)	5 (12.5)	NR
Infections and infestations							
COVID-19	NR	NR	NR	NR	NR	NR	9 (37.5)
Grade 3/4 TEAEs							
Skin disorders							
Rash	NR	NR	1 (1.6)	0 (0.0)	1 (1.1)	1 (2.5)	NR
Gastrointestinal disorders							
Diarrhea	NR	1 (2.6)	0 (0.0)	0 (0.0)	0 (0.0)	0 (0.0)	NR
Vomiting	NR	NR	1 (1.6)	0 (0.0)	1 (1.1)	NR	0 (0.0)
General disorders							
Fatigue	NR	1 (2.6)	0 (0.0)	0 (0.0)	0 (0.0)	0 (0.0)	0 (0.0)
Face edema	NR	NR	0 (0.0)	1 (3.3)	NR	0 (0.0)	NR
Investigations							
AST increased	NR	3 (7.7)	6 (9.8)	1 (3.3)	8 (8.8)	5 (12.5)	1 (4.2)
ALT increased	NR	4 (10.3)	6 (9.8)	2 (6.7)	9 (9.9)	8 (20.0)	NR
LDH increased	NR	NR	1 (1.6)	0 (0.0)	NR	0 (0.0)	NR
GGT increased	NR	NR	NR	NR	NR	7 (17.5)	NR
ALP increased	NR	NR	4 (6.6)	1 (3.3)	NR	0 (0.0)	NR
CPK increased	NR	NR	NR	NR	NR	1 (2.5)	4 (16.7)
CB increased	NR	NR	NR	NR	NR	2 (5.0)	NR
Blood bilirubin increased	NR	NR	NR	NR	NR	2 (5.0)	NR
Lymphocyte count decreased	NR	NR	NR	NR	NR	1 (2.5)	NR
Nervous system disorders							
Dizziness	NR	NR	1 (1.6)	0 (0.0)	NR	0 (0.0)	NR
Musculoskeletal disorders							
Arthralgia	NR	NR	2 (3.3)	0 (0.0)	2 (2.2)	1 (2.5)	0 (0.0)
Eye disorders							
Periorbital edema	NR	NR	1 (3.3)	0 (0.0)	1 (1.1)	0 (0.0)	NR
Vascular disorders							
Hypertension	NR	NR	3 (4.9)	2 (6.7)	7 (7.7)	1 (2.5)	NR
Metabolic/nutritional disorders							
Hypophosphatemia	NR	4 (10.3)	NR	NR	NR	NR	NR
Blood and lymphatic system disorders							
Anemia	NR	1 (2.6)	NR	NR	NR	0 (0.0)	0 (0.0)

^a^Part 2 of the ENLIVEN study included patients initially randomized to placebo in part 1 who entered part 2 and received open label pexidartinib treatment;

^b^One death was reported in a patient with advanced, loco-regionally progressing mucosal melanoma receiving pexidartinib 1000 mg/day monotherapy; this was not considered treatment-related;

^c^Cases reported in (≥ 25% of patients in any study. ALP, alkaline phosphatase; ALT, alanine aminotransferase; AST, aspartate aminotransferase; CB, conjugated bilirubin; COVID-19, coronavirus disease 2019; CPK, creatine phosphokinase; GGT, gamma-glutamyl transferase; LDH, lactate dehydrogenase; NR, not reported; TEAE, treatment-emergent adverse event; TGCT, tenosynovial giant cell tumor

The phase 1 extension study of pexidartinib demonstrated systemic exposure proportional to dose; the time to maximum concentration (T_max_) is relatively short at 1–2 h, with a half-life (t_1/2_) of 16.8 h [51]. No clinically meaningful differences in pexidartinib pharmacokinetics have been identified based on age, sex, race, or mild hepatic impairment [56]; however, exposure is 30% greater in patients with renal impairment (creatinine clearance [CrCL] 15–89 mL/min) than in those with normal renal function (CrCL ≥ 90 mL/min) [14, 57]. The recommended dose is therefore lower for patients with renal impairment (125 mg in the morning and 250 mg in the evening with a low-fat meal) than for those with normal renal function (250 mg twice daily) [57].

### Phase 3 studies

Building upon these earlier-stage clinical trials, the pivotal, two-part, phase 3, multicenter, randomized, placebo-controlled ENLIVEN trial evaluated the efficacy and safety of pexidartinib in patients with symptomatic TGCT for whom surgery was not recommended [38]. Patients were randomized 1:1 to receive pexidartinib or placebo to Week 25 (part one, double-blind), after which all patients received pexidartinib treatment (part two, open-label). The ORR at the end of the double-blind period (Week 25) was significantly improved with pexidartinib versus placebo (39% [n = 61] vs. 0% [n = 59]; 95% CI for difference: 27–52%; p < 0.0001) as evaluated by RECIST v1.1, and by TVS (56% vs. 0%; 95% CI: 42–68%; p < 0.0001; Table 1) [38]. At the data cutoff of January 31, 2018, with a median follow-up of 22 months, the ORR was 53% by RECIST, and 64% by TVS. The most common TEAEs were hair color changes (67.2%), fatigue (54.1%), AST increased (39.3%), nausea (37.7%), and ALT increased (27.9%; Table 2) [38]. Emergence of mixed/cholestatic hepatotoxicity caused the data monitoring committee to stop enrolment six patients short of target [38]; careful monitoring of liver function is therefore advised within two months of treatment initiation [48]. Treatment discontinuation was reported in 13% (8/61) of patients, the majority of which (7/8) were related to hepatic TEAEs [38]. In part two, 30 patients received placebo in part one followed by a crossover, open-label pexidartinib treatment for 25 weeks: the ORR was 30% by RECIST at Week 25 (57% by TVS), increasing to 53% by RECIST (67% by TVS) at data cutoff [38]. Fewer cases of liver enzyme elevation were observed among crossover patients versus those in part one, with no signs of drug-induced cholestatic hepatotoxicity (Table 2).

A single-arm, phase 3, bridging study (A303; NCT04488822) was conducted in the Chinese mainland and Taiwan (China) assessing the efficacy and safety of pexidartinib in Chinese patients with TGCT [58]. At Week 25 (primary endpoint), the ORR by RECIST was 22.5% (47.5% by TVS; Table 1) [58]. Responses were sustained and improved over time to Week 37, with an ORR by RECIST of 35.0% (45.0% by TVS). Among 40 patients, the most common TEAEs included hair color changes (77.5%), increased ALT (60.0%) and AST (57.5%; Table 2) [58]. Grade ≥ 3 TEAEs were reported in 16 (40.0%) of patients, none of which were grade 5. Of 16 patients with AST/ALT ≥ 3 × upper limit of normal (ULN), the majority of events occurred within the first two treatment cycles. Hepatotoxicity was well managed with monitoring of liver function and dose modifications.

### Indication and dosing

**Fig. 1:**
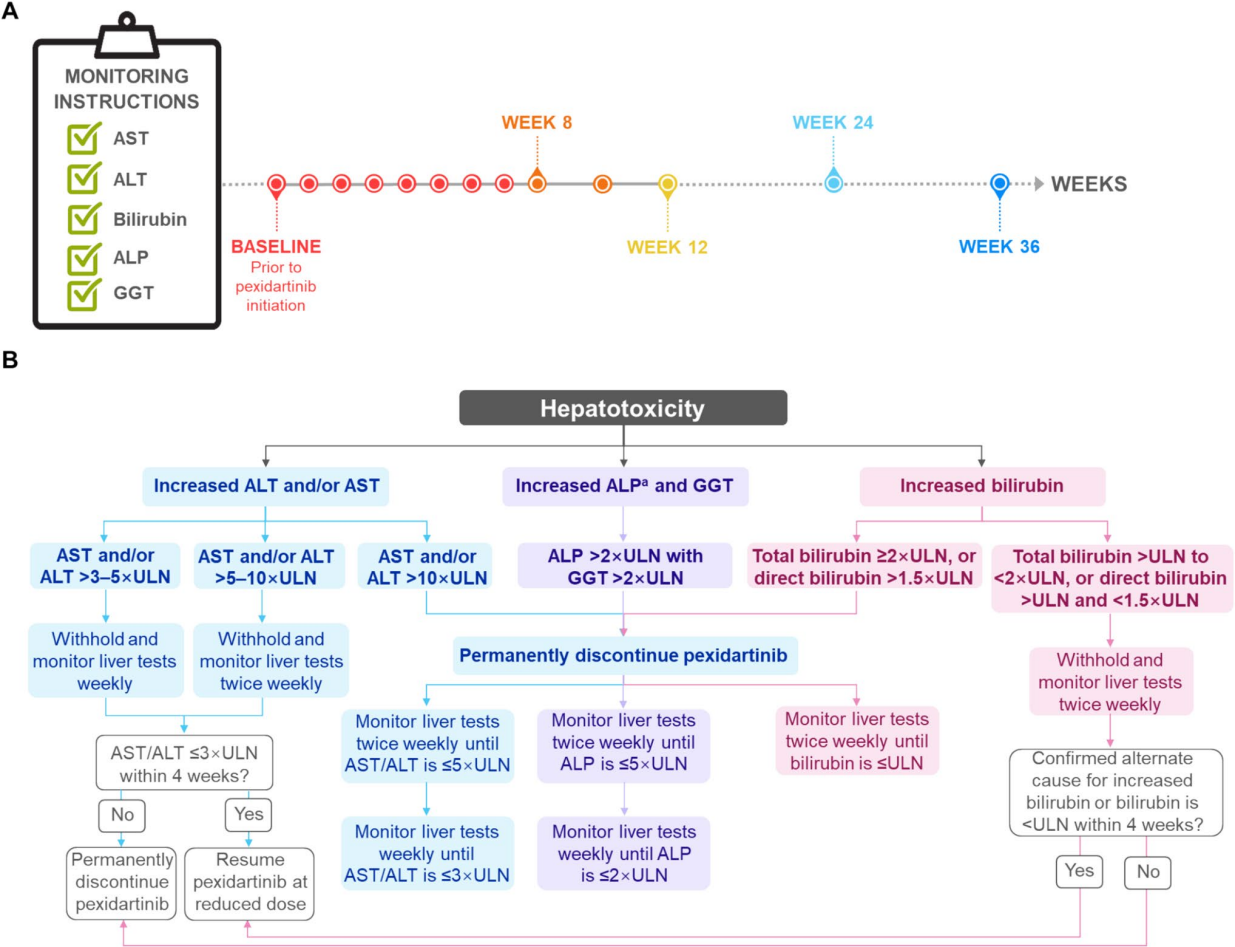
Recommended liver chemistry monitoring **A** and management of hepatotoxicity **B** with pexidartinib treatment. Based on US FDA prescribing guidelines for pexidartinib [57]. ^a^Confirm ALP elevations as liver isozyme fraction. ALP, alkaline phosphatase; ALT, alanine aminotransferase; AST, aspartate aminotransferase; FDA, Food and Drug Administration; GGT, gamma-glutamyl transferase; ULN, upper limit of normal

Based on the results of the ENLIVEN study, pexidartinib was initially approved by the US FDA through a REMS program in 2019 for adult patients with TGCT with severe morbidity or functional impairment who are not amenable to surgery, at a dose of 400 mg twice daily (fasted) [41]; this was since reduced to a recommended dosage of 250 mg twice daily with a low-fat meal, based on food effect analyses [57, 59]. If dose reductions are required for AEs, the first is to 375 mg daily (125 mg in the morning and 250 mg in the evening), and the second to 250 mg daily (125 mg twice daily) with discontinuation advised for patients unable to tolerate the latter [57]. Owing to the risk of embryo-fetal toxicity based on studies conducted in animals, pregnant women should be advised of the potential risk to a fetus, and female patients of reproductive potential are advised to use non-hormonal contraception during and for one month after pexidartinib treatment [57]. Use should be avoided in patients with pre-existing increased serum transaminases or total/direct bilirubin, or active liver or biliary tract disease including increased alkaline phosphatase (ALP), owing to the identified risk of hepatotoxicity with pexidartinib treatment [57]. Pexidartinib use in specific populations is summarized in Table 3, and guidance for the monitoring of liver enzymes and management of hepatotoxicity is summarized in Fig. 1. In terms of clinically important drug interactions, pharmacokinetic studies have identified interactions with moderate or strong CYP3A inducers and acid-reducing agents, found to reduce pexidartinib exposure, while strong or moderate CYP3A inhibitors and glucuronosyltransferase (UGT) inhibitors have been associated with increased pexidartinib exposure [57]. Decreased CYP3A substrate exposure has also been identified with pexidartinib coadministration [57].

**Table 3:** Pexidartinib use in specific populations

Specific population	Risk summary	Recommendation
Pregnant patients	Available human data have not established an associated risk of pexidartinib treatment on birth defects or miscarriage; however, studies conducted in animals have identified an increased risk of post-implantation loss and fetal malformations at doses equivalent to the recommended human exposure at the recommended dose [57]	Pregnant women should be advised on the potential risk to a fetus [57]
Lactating patients	There are no data on the presence of pexidartinib or its metabolites in human or animal milk, and its effects on milk production or in a breastfed child have not been established [57]	Owing to the potential risk of serious AEs in a breastfed child, lactating women should be advised not to breastfeed during and for at least one week after pexidartinib treatment [57]
Patients of reproductive potential	Based on animal studies, pexidartinib has been associated with embryo-fetal toxicity and may impair both male and female fertility, with reductions in pregnancy, viable embryos, and spermatogenic parameters, and increases in post-implantation loss identified in rats [57]	Pregnancy status should be verified prior to pexidartinib initiation. Female patients should be advised to use non-hormonal contraception during and for one month after treatment, and male patients with female partners of reproductive potential advised to use effective contraception during and for one week after treatment [57]
Pediatric patients	Safety and effectiveness has not yet been established in pediatric patients [57]	-
Geriatric patients	Available clinical data have not established whether patients aged ≥ 65 years respond differently from those aged < 65 years [57]	-
Patients with renal impairment	Pexidartinib exposure is approximately 30% greater in patients with mild to severe renal impairment (CrCL 15–89 mL/min) than those with normal renal function (CrCL ≥ 90 mL/min) [57]	Dosage reductions are advised for patients with mild to severe renal impairment (CrCL 15–89 mL/min) [57]
Patients with hepatic impairment	Pexidartinib exposure is 43% increased in patients with moderate hepatic impairment (total bilirubin > 1.5–3 × ULN, not due to Gilbert’s syndrome, with any AST) than those with normal hepatic function (total bilirunib > 3–10 × ULN with AST ≤ ULN); exposure has not been evaluated in patients with severe impairment [57]	Dosage reductions are advised for patients with moderate hepatic impairment; dosage adjustments are not required for those with mild hepatic impairment (total bilirubin ≤ ULN with AST > ULN or total bilirubin > 1–1.5 × ULN with any AST). Pexidartinib use should be avoided in patients with pre-existing hepatic increased serum transaminases, total or direct bilirubin > ULN, or active liver or biliary tract disease [57]

AE, adverse event; AST, aspartate aminotransferase; CrCL, creatinine clearance; ULN, upper limit of normal

### Long-term tumor response

Following the US FDA approval of pexidartinib, subsequent clinical studies have evaluated the long-term effectiveness of pexidartinib treatment. Since the mean age at diagnosis of TGCT is relatively young (< 50 years), patients may require prolonged treatment to support lasting tumor control; it is therefore important to establish long-term tumor control and safety in response to therapeutic options [1]. Final long-term results of the ENLIVEN study, including the 240-week open-label extension period (data cutoff: April 2021), revealed an ORR of 60% by RECIST and 68% by TVS (Table 1), with tumor regrowth occurring in < 25% of responders and median DOR not reached [60, 61]. Similarly, a pooled analysis of three clinical cohorts of pexidartinib for TGCT, including two from the phase 3 ENLIVEN study, reported an ORR of 60% by RECIST (65% by TVS) among patients receiving pexidartinib treatment after a median follow-up of 39 months (32–82 months after first dose; Table 1) [62]. The median DOR was not reached by RECIST, and was 46.8 months by TVS. Together, the increases in ORR from 39% at Week 25 to 60% at Month 39 (median) and Week 240 identified in both analyses suggest that prolonged treatment with pexidartinib may offer patients sustained responses over time [38, 60, 62].

### Patient-reported functional improvements

In the phase 3 ENLIVEN trial, pexidartinib treatment resulted in improvements in patient-reported outcomes, with significant improvements in Patient-Reported Outcomes Measurement Information System − Physical Function (PROMIS) score (improvement from baseline of 4.1 points with pexidartinib versus a reduction of 0.9 with placebo, p = 0.0019) and worst stiffness (improvement from baseline [reduction] of 2.5 points with pexidartinib versus 0.3 with placebo, p < 0.0001; Table 1) [38]. Improvements were also identified in Pain-30 response (≥ 30% decrease in mean Brief Pain Inventory [BPI] NRS score) with pexidartinib (31% improvement from baseline) versus placebo (15% improvement from baseline; p = 0.032). A subsequent exploratory analysis of the ENLIVEN trial found that pexidartinib provided modest improvements in pain versus placebo (26% vs 10% of patients achieved ≥ 50% improvements in worst pain NRS [p = 0.02]; 31% vs 14% met the threshold for minimal clinically important difference [MCID; p = 0.02]), with a mean change from baseline of − 2.5 versus − 0.3 points at Week 25 (part one, p < 0.001; Table 1) [63, 64]. Improvement in pain score was sustained to Week 50, and was significantly associated with reductions in tumor size and volume (both p < 0.001) [63].

Building upon these findings, improvements in patient-reported outcomes with pexidartinib have also been reported in real-world settings. Two recent web-based surveys were administered to adult patients in the Turalio^®^ REMS program: an initial study in 2021 (first survey) and a follow-up in 2022 (second survey) [41, 65, 66]. Based on the first cross-sectional survey, the majority of patients reported improvements in TGCT symptoms (78.3%) and physical function (77.2%, including worst stiffness NRS [p < 0.05] and worst pain NRS [p < 0.05]; Table 1) compared with before pexidartinib initiation [65]. In the second survey, these patients continued to report improvements in patient-reported physical function, stiffness, and pain [67]. Among 31 patients receiving pexidartinib, the majority (59.0%) reported that their symptoms were “much improved” or “very much improved” with pexidartinib treatment, with improvements in stiffness and worst pain NRS scores of ≥ 3 points [67]. At 1-year follow up since first dose, there were no significant changes in physical function and pain and stiffness scores, suggesting pexidartinib treatment may offer patients stable, sustained improvements in physical function [67].

Comparable longitudinal results have also been observed among patients in the REMS program who had newly initiated pexidartinib treatment between April 2022 and October 2023: 72.2% (8/11) patients who newly initiated pexidartinib treatment expressed moderately high satisfaction with treatment, with 87.5% (7/8) reporting that their symptoms were “much improved” or “very much improved” since treatment initiation (Table 1) [66]. The majority of patients also reported reductions in stiffness and pain.

## Long-term safety of pexidartinib and management of hepatotoxicity

As a benign tumor that affects a young patient population, long-term medication is needed to achieve sustained symptom control and functional improvement for TGCT [1]. Therefore, establishing the long-term safety profile of treatment for TGCT is of critical importance. Following the phase 3 ENLIVEN trial, subsequent analyses have further assessed the long-term safety profile of pexidartinib in patients with TGCT [48, 62, 68]. An analysis of long-term treatment that included patients enrolled in ENLIVEN completing an additional 26 months of pexidartinib treatment found a generally well-tolerated safety profile, with most AEs mild or moderate in severity (grades 1 or 2), and no new cases of mixed/cholestatic hepatotoxicity observed [62]. While hepatic TEAEs were reported in 95% (133/140) of patients across pooled clinical studies [68], 91% (128/140) were reversible, low-grade, dose-dependent AST/ALT elevations and 4% (5/140) had serious mixed/cholestatic injury, suggesting that long-term pexidartinib use has a predictable effect on hepatic aminotransferases, but an unpredictable risk of serious cholestatic/mixed liver injury [68]. The onset of serious hepatic events occurred only within the first 2 months of treatment, and all five patients recovered within 7 months of pexidartinib discontinuation [68]. Overall, the safety profile did not indicate long‐term hepatotoxicity or any new safety concerns.

Owing to the identified risk of hepatotoxicity with pexidartinib [69, 70], the approval of pexidartinib in the US was conditional on its prescription via a mandatory REMS program [54, 71]. The recently updated 3- and 5-year safety outcomes of patients with TGCT enrolled in the REMS program provide valuable guidance on the long-term safety of pexidartinib in clinical practice. In a recent cross-sectional survey of 228 patients enrolled in the REMS program, 26.5% (22/83) discontinued pexidartinib due to physician suggestion, abnormal laboratory results, TEAEs, symptom improvements, patient taking a break or did not like taking medication every day, or other reasons [65]. Among those who discontinued pexidartinib due to TEAEs, the most common TEAEs were hair color changes, abnormal liver enzyme tests, and fatigue, with a safety profile consistent with the phase 3 ENLIVEN trial [38, 65].

Results of a 3-year retrospective hepatic safety assessment of patients enrolled in the REMS program were consistent with the phase 3 ENLIVEN trial: among 451 patients, hepatic AEs or laboratory abnormalities suggestive of liver injury were reported among 21 (4.7%, all within two months of treatment), with no cases of irreversible liver injury and no new safety signals observed [72]. Consistent results were observed in a recent 5-year hepatic safety assessment [73]. More than 90% of AEs identified were non-serious and well managed [72]. These results support the use of careful monitoring of liver enzymes, paired with early interventions including dose modification within two months of treatment initiation, to mitigate the risk of potential hepatotoxicity [57, 68, 72]. A long-term observational study is ongoing (NCT04635111; Table 4), aiming to evaluate the long-term risk of hepatotoxicity with pexidartinib treatment and identify any underlying mechanisms of hepatotoxicity [74].

**Table 4:** Overview of ongoing clinical studies of pexidartinib

Trial	Phase	Intervention	Study population	Endpoints
NCT04703322 [102]	2	Pexidartinib 800 mg/day	Japanese patients with TGCT	Dose-limiting toxicities, pharmacokinetics, ORR at Week 25 (RECIST, TVS), highest ORR through approximately 1.5 years (RECIST, TVS), DOR through approximately 1.5 years (RECIST, TVS), BPI score, ROM score, PROMIS score, and safety (TEAEs, SAEs)
NCT04635111 [74]	Observational (post-marketing study)	Pexidartinib (≥ 1 dose)	Patients with symptomatic TGCT associated with severe morbidity or functional limitations who are not amenable to surgery	Frequency of hepatic failure after pexidartinib discontinuation, liver test abnormalities, liver transplant, and deaths
NCT02390752 [82, 103]	1	Pexidartinib	Children and young adults with recurrent or refractory solid tumors or leukemia (including TGCT)	Recommended phase 2 dose, pharmacokinetics, safety and tolerability, antitumor activity, patient-reported and functional outcomes, and correlation between immune biomarkers and response

BOR, best overall response; BPI, Brief Pain Inventory; DOR, duration of response; ORR, objective response rate; PROMIS, Patient-Reported Outcomes Measurement Information System − Physical Function scale; RECIST, Response Evaluation Criteria in Solid Tumors; ROM, range of motion; SAE, serious adverse event; TEAE, treatment-emergent adverse event; TGCT, tenosynovial giant cell tumor; TVS, tumor volume score

Overall, TEAEs associated with pexidartinib are typically transient and reversible, with a risk profile often comparing favorably with radiotherapy/surgery [14]. However, clinicians must consider the risk/benefit on an individual-patient basis when assessing pexidartinib suitability, with careful patient selection and monitoring of liver function advised [48, 72].

## Optimizing pexidartinib treatment for patients with TGCT

Following the phase 3 ENLIVEN study, more recent analyses have begun to evaluate the efficacy of pexidartinib across settings beyond those used in clinical trials to date, in order to improve and optimize pexidartinib treatment for real-world patients with TGCT. These include evaluation of the pharmacokinetic impact of food on pexidartinib and between demographic populations, discontinuation and rechallenge with pexidartinib, and use of pexidartinib in the perioperative setting and among pediatric patients with TGCT. Results of these studies may therefore more closely reflect the utility of pexidartinib in real-world settings, and may help inform clinical decision-making.

### Optimizing dosage and administration based on food effect

Pexidartinib pharmacokinetics are influenced by the fat content of meals: a high-fat meal increases pexidartinib absorption by 100% (versus fasted state); a low-fat meal increases absorption by 60% [59]. Owing to the risk of overexposure and potential toxicity when taking pexidartinib with food, a revised dosing regimen is now recommended by the US FDA, following the approval of pexidartinib 125 mg in 2022: from 800 mg/day (fasted) to 500 mg/day with a low-fat meal [41, 57, 59]. Recent studies have found that pexidartinib exposure (steady-state area under the curve values from 0–24 h) was 21% lower among Asian versus non-Asian patients with TGCT; however, the 95% CI was wide owing to the small number of Asian patients included (n = 8) [56].

### Optimizing long-term treatment strategies: discontinuation and rechallenge

Owing to the chronic nature of TGCT that can affect relatively young patients, long-term pexidartinib treatment may be required for long-term disease control [1]. In real-world settings, patients may sometimes discontinue treatment after experiencing a prolonged treatment benefit or for family planning, and may subsequently wish to resume treatment. A single-arm, open-label, phase 4 study aimed to mimic this pattern of real-world use of pexidartinib treatment, evaluating the impact of discontinuation and retreatment on treatment outcomes [75]. The study enrolled 32 patients with TGCT experiencing clinical benefit in one of four prior phase 1 or phase 3 studies investigating pexidartinib, where patients could choose to continue receiving pexidartinib or discontinue with an option to restart treatment. After 24 months of follow-up, 54.5% (6/11) patients in the treatment-free/retreatment group experienced progressive disease (PD), with a median PFS of 22.8 months, while no patients who chose to continue receiving pexidartinib treatment had PD (n = 21; Table 1) [75]. This suggests that pexidartinib treatment can offer sustained efficacy to patients with TGCT with continuous treatment, and is also feasible in those who restart treatment after a treatment-free period [75].

### Perioperative management

**Fig. 2:**
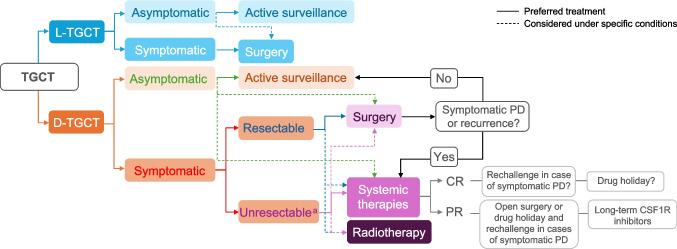
Treatment paradigm for pexidartinib use for patients with TGCT. ^a^Moderate to severe functional impairment, or morbidity associated with surgery. CR, complete response; CSF1R, colony-stimulating factor 1 receptor; D-TGCT, diffuse TGCT; L-TGCT, localized TGCT; TGCT, tenosynovial giant cell tumor; PD, progressive disease; PR, partial response

Following the robust and sustained efficacy demonstrated with pexidartinib as systemic treatment [38, 61], the use of pexidartinib has since been evaluated in the perioperative setting, with case reports showing benefit of using pexidartinib as upfront therapy prior to surgery, and/or as adjuvant treatment [76–78]. While limited in number, these reports suggest that patients with more aggressive disease and structural damage might benefit from a combined systemic/surgical approach [14]. For example, a case study of a patient with TGCT reported sufficient reductions in tumor volume after pexidartinib treatment to avoid a potential surgical resection that was associated with high morbidity (Table 1) [79]. Tumor response was observed within three weeks of pexidartinib treatment, and a 48% reduction in tumor volume after four months of treatment [79]. After 55 months, disease was stable with significant improvements in functional status and pain, and the patient was able to return to work with no surgical intervention. In line with this, use of pexidartinib as upfront therapy was associated with a reduction in tumor volume by ~ 80% in another case study, with corresponding improvements in pain and mobility; the patient was subsequently able to undergo surgery and return to work [77]. Similar findings were reported in several cases in which pexidartinib reduced tumor burden before and after surgery, suggesting that combination strategies could reduce surgical morbidity and the risk of postoperative disease recurrence [76]. Based on this accumulating evidence, a paradigm shift in treatment strategies for patients with TGCT has been proposed (Fig. 2) [78].

In the most recent analysis of the phase 3 registrational ENLIVEN study, three patients successfully underwent surgery after pexidartinib treatment, achieving surgical conversion (Table 1) [80]. This was not possible in the placebo group. This suggests that pexidartinib may have potential as a neoadjuvant therapy, although further research is needed to confirm this.

### Exploring the application of pexidartinib in pediatric TGCT

While pediatric TGCT is rare [81], preliminary data from an ongoing phase 1 study of pediatric patients with TGCT suggest that symptomatic relief and tumor responses can be achieved in a pediatric population (Table 4) [82]. Among three pediatric patients (aged 11–16 years old) with recurrent and/or progressive TGCT and a history of surgery, all achieved significant relief of symptoms within two cycles of pexidartinib treatment (initiated at 500 mg/m^2^/day, interrupted, then resumed and titrated up to symptomatic effect), and returned to normal activities without limitations or pain medication required [82]. Pexidartinib was well tolerated among pediatric patients with TGCT, with no serious AEs reported, and most AEs occurring shortly after administration; the most common AEs were changes in hair color, and grade 1 ALT increased, AST increased, creatine kinase increased, and neutrophil count decreased. Stable dosing based on clinical effect was achieved at 149–214 mg/m^2^/day, offering a potential strategy for pexidartinib use at effective doses below the maximum tolerated dose among patients who may require long-term therapy [82].

With no systemic therapies currently approved for pediatric patients with TGCT, all such prescriptions are currently made off-label [83]. In a real-world analysis of an international observational pediatric patient registry, systemic therapies were received by 17.2% of included patients (21/122), all among patients with diffuse-type (or unknown subtype) TGCT [83]. Among these patients, imatinib was most commonly received (57.1%, 12/21), followed by pexidartinib (38.1%, 8/21) [83].

## Discussion

Following the breakthrough approval of pexidartinib as the first CSF1R inhibitor for TGCT [84], continuous exploration and research are required to further enhance patient benefits, addressing various issues encountered in clinical practice. Since the primary focus of TGCT treatment is to enhance long-term quality of life, there is a need for systemic therapies to place an emphasis on long-term efficacy, the safety and optimization of long-term use, and resistance to such prolonged regimens. To date, just one other systemic treatment, vimseltinib, has been approved for treatment of TGCT [42, 43], with other targeted agents still under investigation [85–88]. The large, long-term accumulated dataset of pexidartinib is therefore invaluable for addressing issues related to prolonged medication use.

Vimseltinib, another CSF1R inhibitor, selectively inhibits CSF1-induced auto-phosphorylation by blocking the switch pocket region of the CSF1R, with an IC_50_ of 0.019 μM, and dose-dependent reductions in nonclassical monocytes with a T_max_ of 1 h and t_1/2_ of 6 days [43, 89, 90], In the phase 3 MOTION study of vimseltinib, the ORR by RECIST was 40%, the majority of TEAEs were grades 1–2 in severity, and the most common grade 3–4 TEAE was increased blood CPK [91]. While the efficacy and safety profiles of pexidartinib and vimseltinib have not been directly compared, the ORR was approximately 40% in both the pivotal phase 3 ENLIVEN and MOTION studies [38, 91], and both were generally well tolerated, with no reports of cholestatic hepatotoxicity or drug induced liver injury in the MOTION trial of vimseltinib [91], and the risk of hepatotoxicity mitigated with liver monitoring and early intervention in the pexidartinib REMS program [72].

Real-world studies have illustrated that long-term pexidartinib treatment is well tolerated and associated with sustained tumor response, an important consideration for many patients requiring prolonged treatment [1, 62]. However, for certain issues, such as whether treatment can be discontinued after achieving complete remission or if rechallenge is possible after recurrence following drug discontinuation, answers have been limited by small sample sizes and study design, necessitating larger studies to reflect drug persistence in clinical use. Additionally, questions such as the criteria for treatment discontinuation, optimal medication cycles, and recurrence rates following discontinuation remain unresolved and require further investigation. Furthermore, the potential issue of resistance to CSF1R inhibitors with long-term use requires close attention. Data on resistance are scarce and few studies have characterized the mechanisms of resistance to these drugs; further understanding will therefore help shape the strategies for managing this [92].

Surgery has long been, and will remain, the most critical treatment modality for TGCT. Therefore, determining how to combine systemic therapy with surgical treatment to maximize patient benefits is important. Currently, evidence in this area is limited to case reports; nevertheless, this may indicate a new direction for evolving treatment paradigms for TGCT, with future studies needed to clarify the role of pexidartinib in combination with surgery [48, 76]. Many practical clinical issues remain unresolved, such as the dosage and duration of preoperative treatments, timing of surgery, the proportion of patients who can become eligible for surgery following systemic therapy, and the impact on recurrence. Addressing these issues will require larger studies.

The most common AE associated with pexidartinib treatment for TGCT across studies was change in hair color [38, 51, 54, 58, 60, 75], an event commonly associated with c-KIT inhibition [93]. As a dual inhibitor of both CSF1R and c-KIT [51], changes in hair pigmentation may be due to its effect on c-KIT; indeed, changes in hair color remain one of the most common TEAEs with pexidartinib treatment among patients with tumors other than TGCT [54, 94, 95]. CSF1R inhibition is undoubtedly the primary mechanism of action of pexidartinb in treating TGCT, and while there is currently no conclusive evidence as to the role of c-KIT inhibition for TGCT, studies evaluating the efficacy of pexidartinib and combination therapy for KIT-mutated solid tumors have been completed, with results pending [96–98].

Aside from managing the common AEs associated with tyrosine kinase inhibitors (TKIs), the management of hepatic AEs is of particular importance for CSF1R inhibitors used to treat TGCT [41]. In clinical practice, pexidartinib use necessitates strict compliance with its REMS program, involving close monitoring of liver-related indicators [41]. Elevated liver enzymes such as AST and ALT have also been reported with other drugs in the same class [70, 99]; however, pexidartinib currently provides more comprehensive and longer-term safety data, making its contributions to managing the long-term safety of this drug class uniquely valuable. Elevated levels of such liver enzymes are commonly used indicators of liver injury or hepatic AEs, with pexidartinib dose modifications recommended based on abnormal values (Fig. 1). Continued attention to longer-term safety results for other CSF1R inhibitors is warranted. Of note, the mechanisms underlying hepatic AEs are still under investigation. Ongoing studies are further assessing long-term hepatic risk and the mechanisms of liver injury in a real-world setting, aiming to mitigate this risk over a long-term treatment period [74].

Further studies focusing on efficacy and safety data across demographic factors, baseline disease presentations, and comorbidity profiles are warranted [14]. Indeed, ongoing clinical studies are evaluating pexidartinib efficacy and safety in Asian patients and children with TGCT (Table 4). Finally, combined therapeutic strategies targeting both CSF1 and other signaling that drives growth of TGCT, such as pathways involved in neoplastic cell interactions, are also of interest for future investigation [100]. Together, these results will help optimize treatment strategies for patients with TGCT, and contribute to improving patient prognosis and quality of life.

## Data Availability

Not applicable.
